# Efficacy and safety of single-anastomosis gastric bypass variants versus sleeve gastrectomy or Roux-en-Y gastric bypass: a systematic review and meta-analysis

**DOI:** 10.1007/s13304-025-02518-1

**Published:** 2026-01-28

**Authors:** Cheng Cheng Jin, Peng Zhang, Chun Gao, Sheng Zhang

**Affiliations:** Department of Gastrointestinal Surgery, Tongji Hospital, Tongji Medical College, Huazhong University of Science and Technology, Wuhan, People’s Republic of China

**Keywords:** Single-anastomosis gastric bypass, Transit bipartition, Sleeve gastrectomy, Roux-en-Y gastric bypass, Meta-analysis, Obesity surgery

## Abstract

Single-anastomosis gastric bypass variants, collectively termed sleeve gastrectomy with transit bipartition (SG-TB), have emerged as simplified metabolic procedures. This meta-analysis compares the efficacy and safety of SG-TB against sleeve gastrectomy (SG) and Roux-en-Y gastric bypass (RYGB). We systematically searched multiple databases through September 2025 for comparative studies. Random-effects models were used for meta-analysis, calculating mean differences (MD) and odds ratios (OR) with 95% confidence intervals (CI). Twenty studies were included. Compared to SG, SG-TB resulted in significantly greater 1-year total weight loss (TWL MD = 3.5%, CI 1.5–5.5%) and excess weight loss (EWL MD = 10.8%, CI 8.6–13.0%). For type 2 diabetes remission, SG-TB showed a positive trend but with substantial uncertainty (OR = 2.74, CI 0.61–12.32). SG-TB demonstrated significantly higher GERD remission rates versus SG (OR = 6.90, CI 1.98–24.09). Weight loss and diabetes remission were comparable between SG-TB and RYGB. Nutritional assessment revealed generally comparable profiles, with SG-TB showing better iron/vitamin B12 preservation versus RYGB but lower vitamin D levels versus SG. SG-TB had a comparable short-term safety profile to both SG and RYGB, with no significant differences in perioperative complications, despite a longer operative time versus SG. SG-TB procedures offer superior weight loss compared to SG and achieve metabolic outcomes equivalent to RYGB, with a comparable short-term safety profile. The substantial uncertainty in diabetes remission estimates against SG warrants cautious interpretation. These findings position SG-TB as a compelling and effective surgical option for severe obesity and related metabolic disease.

**Supplementary Information:**

The online version contains supplementary material available at 10.1007/s13304-025-02518-1.

## Introduction

Obesity represents a critical global health challenge, with its prevalence escalating worldwide and imposing a substantial burden on healthcare systems due to its strong association with type 2 diabetes mellitus (T2DM), hypertension, dyslipidemia, and other cardiometabolic disorders [1–3]. Metabolic and bariatric surgery (MBS) remains the most effective intervention for achieving significant and sustained weight loss and comorbidity remission [4–8]. Among the various techniques, Roux-en-Y gastric bypass (RYGB) and sleeve gastrectomy (SG) have been widely adopted and are considered gold standards in many settings [9, 10]. However, long-term weight recurrence and comorbidity relapse remain significant concerns, particularly in super-obese populations [11–14].

In recent years, several single-anastomosis procedures have emerged as technically simplified and metabolically potent alternatives. These include single anastomosis sleeve ileal (SASI) bypass, single anastomosis sleeve jejunal (SASJ) bypass, and their variants with Braun anastomosis (SASI-B), collectively referred to as stomach intestinal pylorus-sparing (SIPS) or SG with transit bipartition (SG-TB) procedures [15, 16]. These procedures share a common principle: combining a sleeve gastrectomy with a single gastro-enteric anastomosis, typically 250–300 cm from the ileocecal valve, to create a ‘bipartition’ of nutrient flow. This design aims to enhance metabolic outcomes through expedited nutrient delivery to the distal intestine while preserving partial physiological transit through the duodenum, potentially offering a favorable balance between metabolic efficacy and nutritional safety compared to more extensive malabsorptive procedures [17–20].

Preliminary studies suggest that these procedures may offer superior weight loss and T2DM remission compared to SG and similar or better outcomes than RYGB, with a favorable safety profile [21, 22]. However, the evidence remains fragmented, derived predominantly from small, heterogeneous single-center series with limited follow-up. A comprehensive synthesis comparing the efficacy and safety of these novel single-anastomosis techniques against established procedures is lacking.

Therefore, we conducted this systematic review and meta-analysis to quantitatively compare the outcomes of SG-TB procedures against both SG and RYGB. We focused on weight loss metrics, rates of T2DM and hypertension remission, and both short- and long-term complication profiles. This analysis aims to provide clinicians and policymakers with high-level evidence to inform surgical decision-making for patients with severe obesity and related metabolic diseases.

## Methods

### Study protocol and search strategy

We conducted a systematic literature search following the Cochrane Handbook, PRISMA, and MOOSE (Meta-analysis Of Observational Studies in Epidemiology) guidelines [23, 24]. We conducted this systematic review and meta-analysis in accordance with the methodological standards outlined in the Cochrane Handbook for Systematic Reviews of Interventions [25]. Electronic databases–PubMed, Cochrane Library, Embase and Scopus–were queried from inception through September 1st, 2025, for English-language studies comparing single-anastomosis procedures (SASI, SASJ, SASI-B, or other SG-TB variants) with either sleeve gastrectomy (SG) or Roux-en-Y gastric bypass (RYGB).

The search strategy combined terms for the interventions and outcomes using Boolean operators. Key search terms included: Procedure terms: “single anastomosis sleeve ileal”, “SASI”, “SASJ”, “SASI-B”, “stomach intestinal pylorus sparing”, “SIPS”, “transit bipartition”, “SG-TB”, “single anastomosis duodeno-ileal”, “SADI-S”; Comparator terms: “sleeve gastrectomy”, “SG”, “Roux-en-Y gastric bypass”, “RYGB”, “gastric bypass”; Outcome terms: “weight loss”, “TWL”, “EWL”, “diabetes remission”, “complications”, “safety”.

We explored all relevant keyword combinations and supplemented the electronic search by manually reviewing reference lists of included articles and relevant reviews. The study protocol was registered in PROSPERO (CRD420251164312).

### Study selection

We included randomized controlled trials, prospective or retrospective cohort studies, and matched comparative studies that directly compared SG-TB procedures (SASI, SASJ, SASI-B, or related SG-TB variants) with either SG or RYGB. We excluded reviews, editorials, case reports, and previous meta-analyses.

Studies qualified for inclusion if they met all following criteria: (1) compared SG-TB procedures with SG or RYGB as primary bariatric interventions; (2) reported weight loss outcomes as total weight loss (TWL), excess weight loss (EWL), or BMI change; (3) provided data on comorbidity remission, specifically type 2 diabetes (with pre- and postoperative HbA1c), dyslipidemia, or obstructive sleep apnea; (4) documented postoperative complications, operative time, or hospital stay. The population exclusion criteria for this review are as follows: We will exclude studies that are not comparative in nature, specifically reviews, editorials, case reports, and previous meta-analyses. Furthermore, we will exclude any primary study that does not directly compare a single-anastomosis SG-TB procedure (e.g., SASI, SASJ, SASI-B) with either sleeve gastrectomy (SG) or Roux-en-Y gastric bypass (RYGB) as the primary surgical intervention for obesity. Studies that do not report quantitative data on at least one of our predefined outcomes of interest (e.g., weight loss, comorbidity remission, complications) will also be excluded.

Two investigators (C.C.J and S.Z) independently screened titles and abstracts, then performed blinded full-text reviews. We resolved disagreements through consensus and documented all exclusion reasons systematically.

### Data extraction and outcome measurement

We systematically extracted baseline characteristics, including preoperative BMI, age, gender distribution, follow-up duration and outcome measurements, for summary in Table 1. Outcome measurements included: (1) Weight loss outcomes: TWL: percentage loss from baseline weight; EWL: percentage loss relative to excess weight above ideal body weight; (2) Comorbidity remission: We extracted remission rates for T2DM, hypertension, dyslipidemia, gastroesophageal reflux disease (GERD), and obstructive sleep apnea syndrome (OSAS) as specifically defined and reported in each primary study. All definitions and measurement timepoints were documented verbatim and summarized. GERD assessment methods varied across studies, including symptom-based questionnaires, endoscopic evaluation, and medication usage documentation. (3) Surgical characteristics: perioperative complications within 30 days, operative time, length of hospital stay; (4) Nutritional and symptom outcomes: nutritional parameters: hemoglobin, albumin, iron, calcium, vitamin B12, and vitamin D levels, nutritional deficiency status: anemia, hypoalbuminemia, vitamin deficiencies, etc. symptom prevalence: vomiting, GERD, constipation, diarrhea.

**Table 1:** Characteristics of studies included in meta analysis

Author	Year	Country	Study design	Patients, *n*	Age (mean, yrs)	BMI (mean, kg/m^2^)	Follow-up, month	Outcomes
Qin [26]	2025	China	Prospective	35 SASI, 70 SG	37.1 37.9	35.0 ± 5.5 36.9 ± 4.6	12	Weight comorbidities Complication
Wael [27]	2025	Egypt	Retrospective	33 SASI, BMI > 60 40 RYGB, BMI > 60	45.2 48.2	63.3 ± 1.6 63.5 ± 2.7	12	Weight comorbidities Complication
Erol [28]	2024	Turkey	Retrospective	51 SASI, T2DM 68 RYGB, T2DM	42.5 45.5	42.8 ± 7.5 44.6 ± 7.6	24	Weight comorbidities
Foschi [29]	2024	Italy	Retrospective	20, SASI, T2DM 20, SG, T2DM 20 SADI-S, T2DM	53 50 52	42 ± 1.7 43 ± 1.7 42 ± 1.8	60	Weight comorbidities Complication
Yu [30]	2024	China	Retrospective	264, SG, MS 30, SASI, MS 30 OAGB, MS	30.1 37.6 34.8	38.7 ± 6.2 39.2 ± 5.1 40.5 ± 4.5	12	Weight comorbidities Complication
Yildirak [31]	2023	Turkey	Retrospective	31, SASI, T2DM 30, SG, T2DM	49.2 48.4	45.9 ± 5.8 46.9 ± 5.0	12	Weight comorbidities
Kirkil [32]	2023	Turkey	Retrospective	111, SG-TB, T2DM 191, SG, T2DM 136, OAGB, T2DM	38 43.6 43.1	38 ± 11.5 43.6 ± 10.3 43.1 ± 10.7	12	Weight Comorbidities
Ece [33]	2021	Turkey	Retrospective	26, SG-TB, T2DM 83 RYGB, T2DM	47.3 50.6	43.8 ± 2.1 45.2 ± 2.6	12	Weight comorbidities Complication
Mahdy [34]	2021	Egypt	Retrospective	46 SASI 46 RYGB	38.4 38.3	44.4 ± 9.8 41.1 ± 8.7	12	Weight comorbidities Complication
Mahdy [35]	2021	Egypt	Retrospective	74, SASI 99, SG 91, OAGB	39 29.6 38.4	42.1 ± 14.5 43.5 ± 5.5 44.8 ± 7.7	12	Weight complication Comorbidities
Madyan [36]	2020	Egypt	Retrospective	20 SASI, 20 SG	35.4 35	53.7 ± 5.9 57.1 ± 13.8	12	Weight Complication Comorbidities
Emile [37]	2020	Egypt	Multi-center Retrospective	58 SASI, T2DM 58 SG, T2DM	37.9 36.9	48.9 ± 16.9 51.5 ± 25.9	12	Weight comorbidities Complication
Yormaz [38]	2017	Turkey	Retrospective	19, SG-TB, T2DM 35, SG, T2DM 29, BPD-DS, T2DM	47.3	37.3 ± 4.3 36.6 ± 2.2 37.4 ± 2.7	12	Weight complication Comorbidities

RCT: randomized clinical trial; BMI, body mass index; T2DM, type 2 diabetes mellitus; SASI, anastomosis sleeve ileal bypass; SASJ, single anastomosis sleeve jejunal bypass, SASI-B, SASI with Braun anastomosis; SG-TB, SG with transit bipartition, RYGB, Roux-en-Y gastric bypass; SG, sleeve gastrectomy; BPD-DS, Biliopancreatic Diversion-Duodenal Switch; OAGB, one anastomosis gastric bypass

The primary objective of this meta-analysis was to compare SG-TB procedures (SASI, SASJ, SASI-B) with sleeve gastrectomy or Roux-en-Y gastric bypass in terms of weight loss efficacy and postoperative safety profiles. Secondary objectives included assessing differences in comorbidity remission–specifically type 2 diabetes, hypertension, dyslipidemia, GERD, and OSAS—as well as evaluating operative time, perioperative complications, and long-term nutritional outcomes.

Two investigators independently extracted data using a standardized form, resolving discrepancies through consensus. We documented all outcome definitions as originally reported in the included studies without imposing uniform criteria for comorbidity remission.

### Statistical analysis

We performed meta-analyses to calculate pooled effect sizes using R version 4.5.1. For continuous outcomes–including TWL, EWL, BMI, operation time, etc. we computed mean differences (MDs) with 95% confidence intervals (CI). For dichotomous variables such as remission rates and complication frequencies, we calculated odds ratios (ORs) with 95% CIs.

All analyses employed random-effects models (restricted maximum likelihood) to account for between-study heterogeneity. We assessed heterogeneity using the I^2^ statistic, considering values < 25% as minimal, 25–50% as low, 50–75% as moderate, and > 75% as substantial. We conducted subgroup analyses and meta-regressions to explore potential sources of heterogeneity when indicated.

We visualized results using forest plots and performed sensitivity analyses to evaluate the robustness of findings. All analytical methods ensured reproducibility and transparency in reporting.

### Quality assessment and publication bias

Two investigators independently assessed study quality using the Newcastle-Ottawa Scale (NOS) for cohort studies. The NOS evaluates eight items across three domains: participant selection, study group comparability, and outcome assessment. We classified studies scoring ≥ 7 as high quality, 4–6 as moderate, and < 4 as low quality, resolving discrepancies through consensus [39] (Supplemental Table 9).

We assessed publication bias using funnel plots and Egger’s regression test [40, 41], with *p* < 0.10 indicating statistical significance. The certainty of the evidence for each study was evaluated using the Grading of Recommendations, Assessment, Development, and Evaluation (GRADE) approach [42]. All analyses employed the metafor package in R, ensuring comprehensive evaluation of potential small-study effects.

## Results

### Study selection and characteristics

**Fig. 1:**
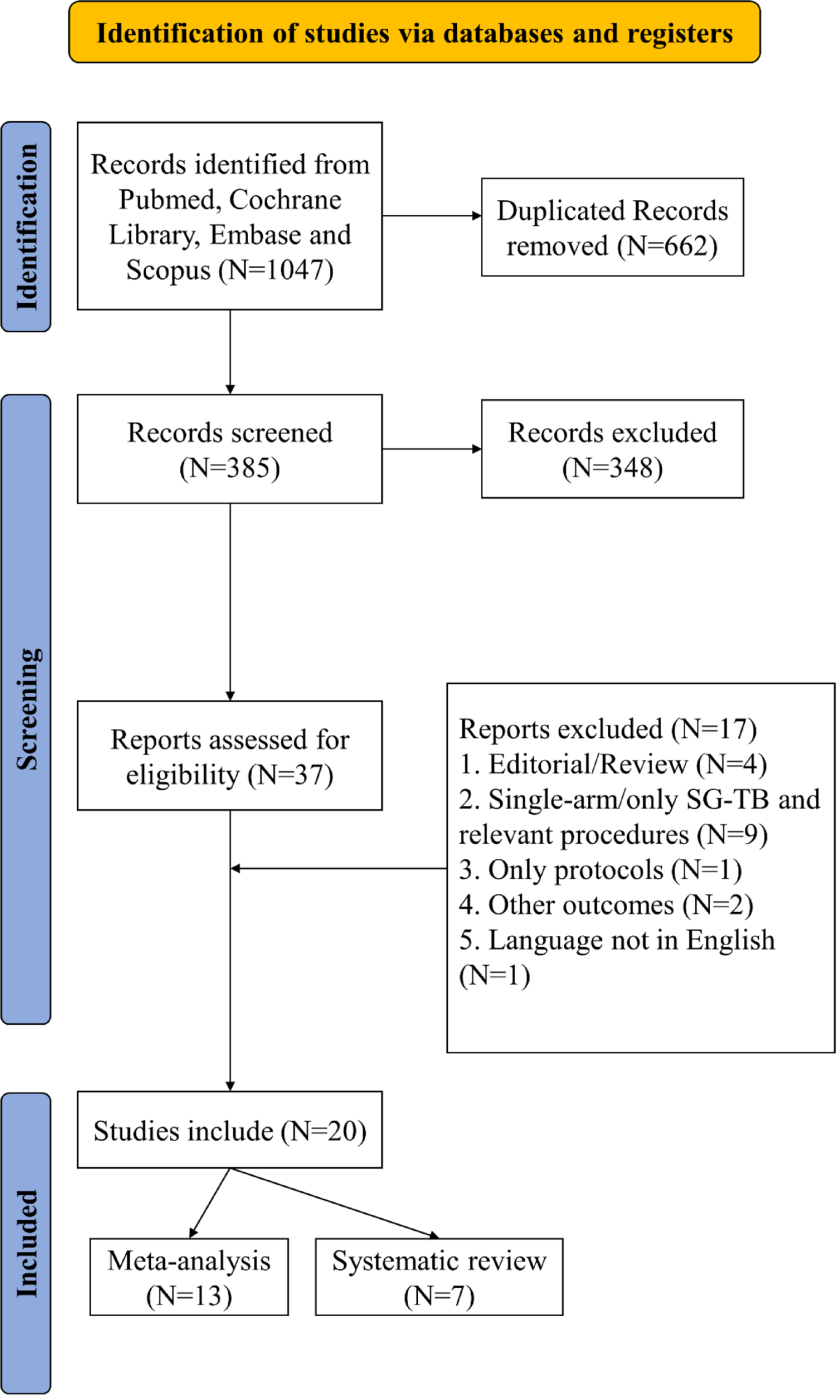
PRISMA flow diagram of study selection

Our systematic search identified 385 records, from which we selected 37 articles for full-text review. Twenty studies met the inclusion criteria for systematic review [26–38, 43–49], with thirteen providing sufficient data for meta-analysis [26–38]. Figure 1 illustrates the study selection process.

The analyzed studies enrolled between 22 and 438 participants. Nine studies compared SG-TB procedures with sleeve gastrectomy [26, 29–32, 35–38], while four evaluated SG-TB against Roux-en-Y gastric bypass [27, 28, 33, 34]. Table 1 summarizes the detailed characteristics of all included studies. The 13 studies included in meta-analysis reported follow-up durations of 1 year (11 studies) [26, 27, 30–38], 2 years (1 study) [28], and 5 years (1 study) [29]. We extracted comorbidity remission data for type 2 diabetes (13 studies) [26–38], hypertension (9 studies) [26–28, 30, 33–37], and dyslipidemia (7 studies) [26–28, 30, 34, 35, 37]. We additionally summarize operative times and perioperative complication rates across all comparative studies. The summary of above outcomes was shown in Table 2.

**Table 2:** Summary of efficacy and safety of SG-TB surgery compared with SG or RYGB

Author (ref)	Study design	Follow-up	1 year %EWL	%TWL	Resolved % T2DM	Hypertension	Dyslipidemia	Perioperative complication	Operation time (mins)
Qin [26]	SASI SG	1 year	NR NR	37 29.9	90% 50%	80% 84.6%	96.4% 93.3%	14.2% 8.6%	140.3 ± 22.8 114.9 ± 21.6
Wael [27]	SASI RYGB	1 year	33.7 29.3	20.4 17.7	88.9% 87.5%	95% 100%	100% 100%	21.2% 20.0%	82.3 ± 30.3 145.2 ± 41.3
Erol [28]	SASI RYGB	2 year	96.7 90	39 36.9	96.1% 95.6%	97.1% 98%	100% 100%	2% 0%	148.4 ± 23.1 155.5 ± 28.6
Foschi [29]	SASI SG	5 year	77.5 65	NR NR	80% 30%	NR NR	NR NR	5% 5%	NR NR
Yu [30]	SASI SG	1 year	88.2 74.9	27.7 26.5	83.3% 63.9%	87.5% 75%	76.5% 62.6%	NR NR	NR NR
Yildirak [31]	SASI SG	1 year	71.7 69.7	32.4 31.9	32.3% 46.7%	NR NR	NR NR	NR NR	NR NR
Kirkil [32]	SG-TB SG	1 year	98.3 89	NR NR	76.6% 95.3%	NR NR	NR NR	NR NR	NR NR
Ece [33]	SG-TB RYGB	1 year	29.7 30.9	68.4 71.3	77% 79.6%	36.8% 38.3%	NR NR	11.4% 14.4	NR NR
Mahdy [34]	SASI RYGB	1 year	78.5 79.4	30.4 33.4	82.7% 73.7%	57.1% 58.3%	76.9% 100%	13% 4.3%	NR NR
Mahdy [35]	SASI SG	1 year	87.6 72.5	36.1 31.6	97.7% 71.4%	75% 64.3%	76.9% 57.1%	4% 0%	NR NR
Madyan [36]	SASI SG	1 year	65.2 57.4	NR NR	100% 100%	75% 66.7%	NR NR	10% 10%	97.7 ± 11.7 97.7 ± 15.3
Emile [37]	SASI SG	1 year	72.6 60.4	37.8 33.3	95.8% 70%	57.1% 62.5%	87.5% 66.7%	6.9% 20.7%	108.7 ± 14.7 92.8 ± 14.6
Yormaz [38]	SG-TB SG	1 year	NR NR	27.1 23.2	82.9% 35.3%	NR NR	NR NR	0% 0%	139.2 ± 41.4 50.4 ± 14.4

T2DM, type 2 diabetes mellitus; SASI, anastomosis sleeve ileal bypass; SASJ, single anastomosis sleeve jejunal bypass, SASI-B, SASI with Braun anastomosis; SG-TB, SG with transit bipartition, RYGB, Roux-en-Y gastric bypass; SG, sleeve gastrectomy; BPD-DS, Biliopancreatic Diversion-Duodenal Switch; EWL, excess weight loss; TWL, total weight loss; NR, not reported

Supplemental Tables 1 and 2 summarized the relevant information of the studies not included in the meta-analysis. Complete weight loss trajectories for all 20 systematically reviewed studies appear in Supplemental Table 3, while detailed metabolic parameters–including HbA1c, lipid profiles, GERD, and OSAS outcomes–are comprehensively documented in Supplemental Table 4.

### Weight loss outcome

**Fig. 2:**
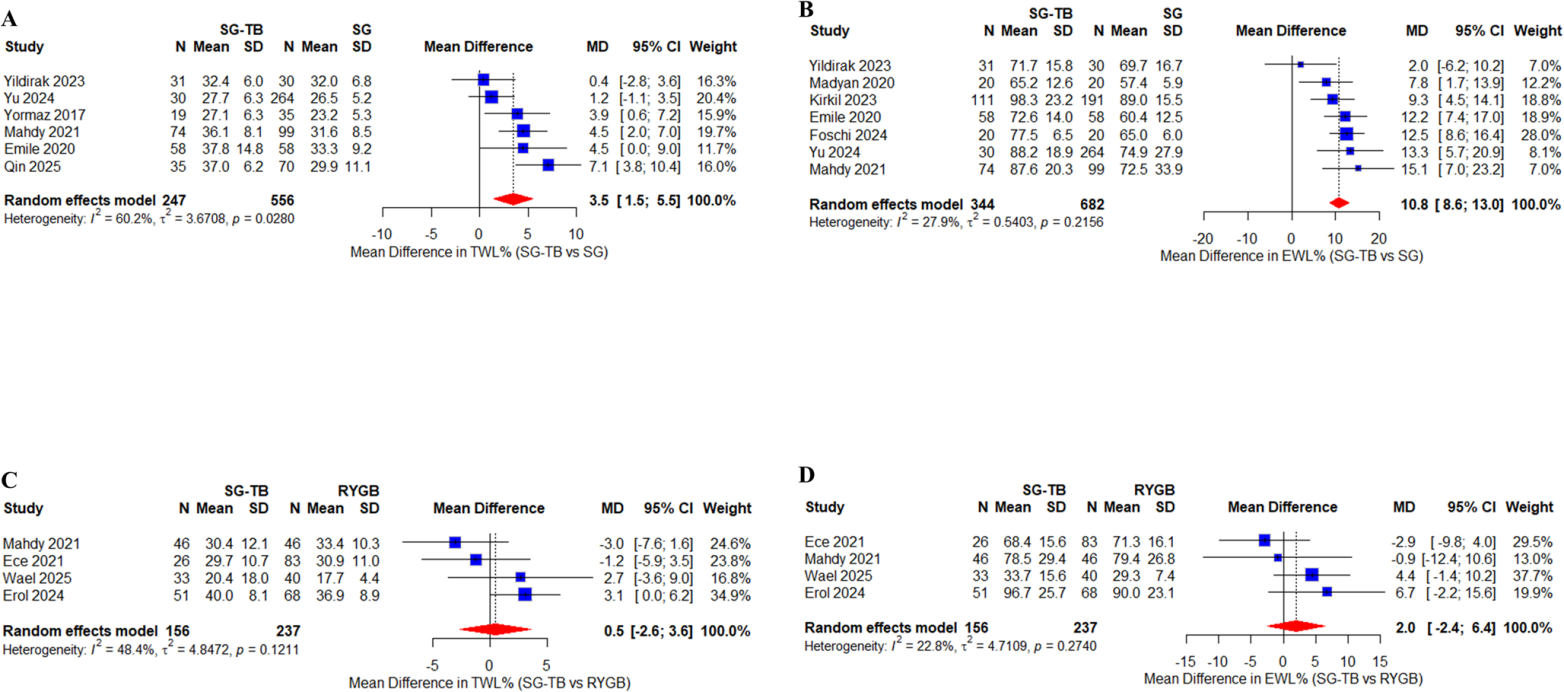
Forest plots of weight loss outcomes at 1 year: SG-TB vs. SG and RYGB. **A** Total weight loss (TWL, %) comparing SG-TB with SG. **B** Excess weight loss (EWL, %) comparing SG-TB with SG. **C** Total weight loss (TWL, %) comparing SG-TB with RYGB. **D** Excess weight loss (EWL, %) comparing SG-TB with RYGB. All analyses were performed using a random-effects model. Mean differences (MD) with 95% confidence intervals (CI) are shown. Heterogeneity was assessed using the I^2^ statistic

Six studies (*n* = 803) [26, 30, 31, 35, 37, 38], demonstrated significantly greater 1-year TWL with SG-TB versus SG (MD = 3.5%, 95% CI 1.5–5.5%, *p* = 0.0013), despite moderate-high heterogeneity (I^2^ = 60.2%) (Fig. 2A). Seven studies (*n* = 1026) [29–32, 35–37] consistently showed superior EWL for SG-TB (MD = 10.8%, 95% CI 8.6–13.0%, *p* = 0.0005) with low-moderate heterogeneity (I^2^ = 27.9%) (Fig. 2B). Both analyses revealed no publication bias and maintained significance in sensitivity analyses (Table 3).

**Table 3:** Summary of key findings

Outcome	Compare	Measurement	Include study	MD or OR (95% CI)	*p* value	I^2^ value	Egger’s *p* value	Conclusion	Certainty of evidence
Weight loss	SG-TB SG	1 year TWL%	6 (*N* = 803)	MD = 3.5% (1.5–5.5%)	0.0013	60.2%	0.756	SG-TB showed significantly greater 1 year TWL% than SG, with moderate to high heterogeneity but consistent findings in sensitivity test and no publication bias	Low 1, 2
	SG-TB SG	1 year EWL%	7 (*N* = 1026)	MD = 10.8% (8.6–13.0%)	0.0005	27.9%	0.324	SG-TB demonstrated significantly superior 1 year EWL% compared to SG, with low heterogeneity but robust sensitivity analysis and no significant publication bias	Low 1
	SG-TB RYGB	1 year TWL%	4 (*N* = 393)	MD = 0.5% (−2.6-3.6%)	0.89	48.4%	0.537	No significant difference in 1 year TWL% was found between SG-TB and RYGB, with moderate heterogeneity and consistent findings across sensitivity analyses	Low 1, 2
	SG-TB RYGB	1 year EWL%	4 (*N* = 393)	MD = 2.0% (−2.4-6.4%)	0.81	22.8%	0.537	SG-TB and RYGB demonstrated comparable efficacy in 1 year EWL%, with low heterogeneity indicating consistent results across studies	Low 1
Diabetes remission	SG-TB SG	1 year CR	8 (*N* = 660)	OR = 2.74 (0.61–12.32)	0.19	87%	0.01	SG-TB showed a non-significant trend toward higher diabetes remission compared to SG at 1-year follow-up, with high heterogeneity and potential publication bias among the included studies	Low 1, 2, 3
	SG-TB RYGB	1 year CR	4 (*N* = 301)	OR = 1.11 (0.53–2.31)	0.77	0%	0.63	No significant difference in diabetes remission was found between SG-TB and RYGB, with low heterogeneity indicating consistent results across studies	Low 1
HTN remission	SG-TB SG	1 year CR	5 (*N* = 183)	OR = 1.25 (0.53–2.91)	0.61	0%	0.95	No significant difference in hypertension remission was found between SG-TB and SG, with low heterogeneity indicating consistent results across studies	Low 1
	SG-TB RYGB	1 year CR	4 (*N* = 243)	OR = 0.85 (0.38–1.87)	0.68	0%	0.16	No significant difference in hypertension remission was found between SG-TB and RYGB, with low heterogeneity indicating consistent results across studies	Low 1
Dyslipidemia remission	SG-TB SG	1 year CR	4 (*N* = 289)	OR = 2.18 (0.92–5.17)	0.07	0%	0.28	SG-TB showed a non-significant trend toward higher dyslipidemia remission rates compared to SG, with low heterogeneity indicating consistent results across studies	Low 1, 5
GERD remission	SG-TB SG	1 year CR	4 (*N* = 86)	OR = 6.90 (1.98–24.09)	0.002	0%	0.46	SG-TB demonstrated significantly higher GERD remission rates compared to SG at 1-year follow-up, with no heterogeneity and no evidence of publication bias	Very low 1, 2, 5
Complication	SG-TB SG	Postoperative	6 (*N* = 528)	OR = 1.03 (0.34–3.07)	0.96	44%	0.36	No significant difference in postoperative complication rates was found between SG-TB and SG, with moderate heterogeneity among the included studies	Low 1, 2
	SG-TB RYGB	Postoperative	4 (*N* = 392)	OR = 1.32 (0.62–2.78)	0.47	0%	0.31	No significant difference in postoperative complication rates was found between SG-TB and RYGB, with low heterogeneity indicating consistent results across studies	Low 1

OR, odd ratio; CR, complete remission; MD, mean difference; CI, confidence interval; T2DM, type 2 diabetes mellitus; SG-TB, SG with transit bipartition, RYGB, Roux-en-Y gastric bypass; SG, sleeve gastrectomy; EWL, excess weight loss; TWL, total weight loss; NR, not reported

GRADE downgrading reasons:

1. Downgraded one point for risk of bias: due to moderate or high risk of bias associated with study design (likely observational), attrition, measurement of outcomes, and statistical analysis in the included studies.

2. Downgraded one point for inconsistency: due to substantial or moderate statistical heterogeneity (I^2^ > 50% or a visually inconsistent forest plot)

3. Downgraded one point for Publication Bias: Indicated by a significant Egger’s test (*p* < 0.10)

4. Downgraded one point because of publication bias

5. Downgraded one point for Imprecision: Because the 95% confidence interval is wide and includes both a potential appreciable benefit and no effect (or appreciable harm), or because of a small total sample size and/or event count

Four studies (*n* = 393) [27, 28, 33, 34] demonstrated comparable weight loss efficacy between SG-TB and RYGB at 1-year follow-up. The pooled analysis showed no significant difference in TWL (MD = 0.5%, 95% CI −2.6–3.6%, *p* = 0.89) with moderate heterogeneity (I^2^ = 48.4%) (Fig. 2C). Similarly, EWL analysis revealed equivalent outcomes (MD = 2.0%, 95% CI −2.4–6.4%, *p* = 0.81) with low heterogeneity (I^2^ = 22.8%) (Fig. 2D). Both analyses showed consistent results across sensitivity testing and no publication bias (Table 3).

### Remission of the comorbidities

**Fig. 3:**
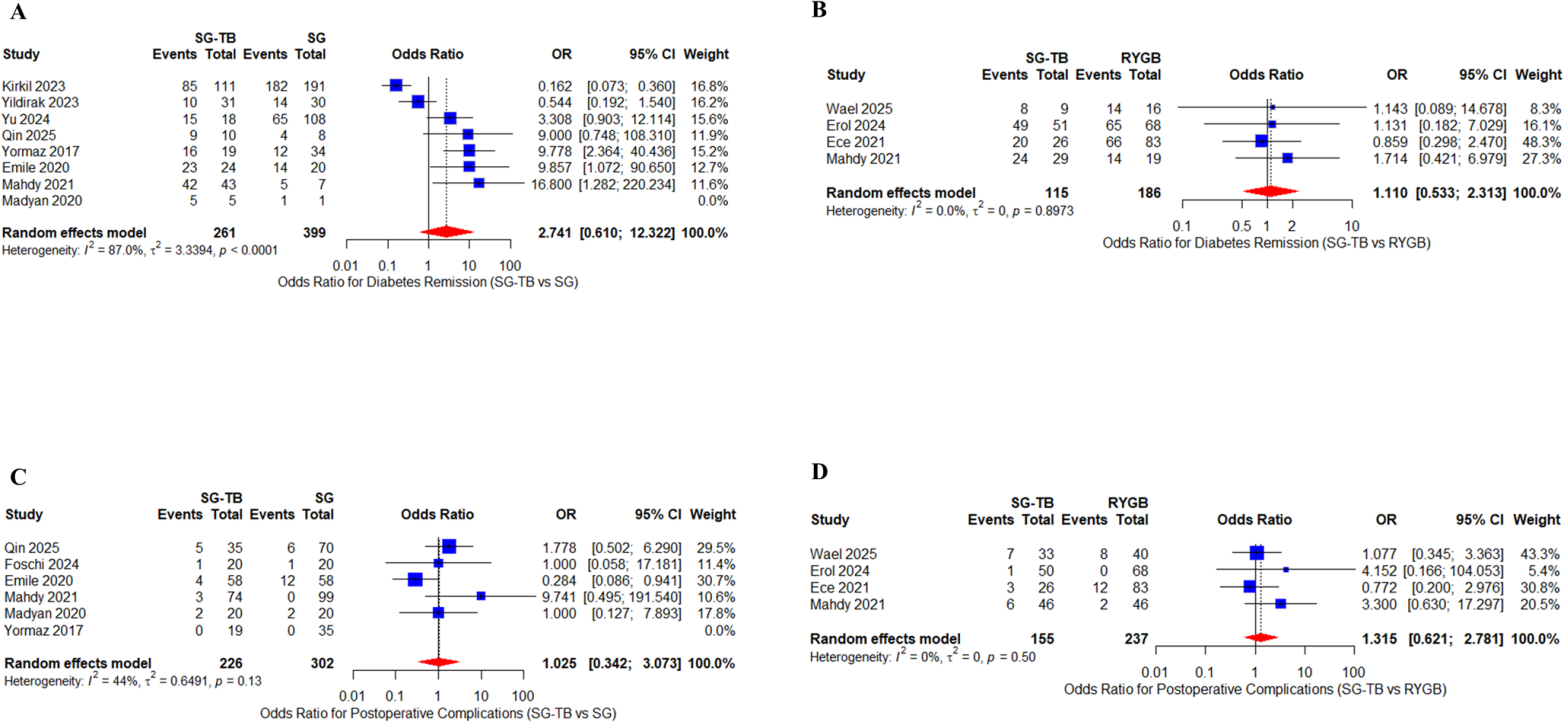
Forest plots of diabetes remission and postoperative complications: SG-TB vs. SG and RYGB. **A** Odds ratios (OR) for type 2 diabetes remission comparing SG-TB with SG. **B** Odds ratios for type 2 diabetes remission comparing SG-TB with RYGB. **C** Odds ratios for postoperative complications comparing SG-TB with SG. **D** Odds ratios for postoperative complications comparing SG-TB with RYGB. All analyses were performed using a random-effects model. Odds ratios with 95% confidence intervals (CI) are shown. Heterogeneity was assessed using the I^2^ statistic

Analysis of T2DM complete remission revealed distinct patterns between comparisons. Eight studies (*n* = 660) [26, 30–32, 35–38] demonstrated a non-significant trend favoring SG-TB over SG (OR = 2.74, 95% CI 0.61–12.32, *p* = 0.19), though with substantial heterogeneity (I^2^ = 87%). In contrast, four studies (*n* = 301) [27, 28, 33, 34] showed comparable diabetes remission rates between SG-TB and RYGB (OR = 1.11, 95% CI 0.53–2.31, *p* = 0.77) with minimal heterogeneity (I^2^ = 0%) (Fig. 3A and B) The SG-TB versus RYGB analysis demonstrated consistent results across sensitivity testing (Table 3).

Detailed T2DM parameters–including FPG, HbA1c, and C-peptide measurements alongside remission definitions–are comprehensively documented in Supplemental Table 5. Patients undergoing SG-TB presented with significantly more advanced diabetic disease compared to SG controls. The SG-TB group had a markedly longer weighted mean diabetes duration (*p* < 0.05), higher baseline HbA1c (*p* < 0.05), and elevated fasting plasma glucose (*p* < 0.05). Additionally, SG-TB patients demonstrated significantly greater insulin use (*p* < 0.05), indicating more impaired β-cell function. In contrast, comparison between SG-TB and RYGB groups demonstrated well-balanced baseline characteristics across all measured parameters including diabetes duration, HbA1c, fasting glucose, and C-peptide levels (all *p* > 0.05), establishing a solid foundation for direct comparison of postoperative metabolic outcomes between these procedures.

Hypertension remission analyses demonstrated comparable outcomes across procedures. Five studies (*n* = 183) [26, 30, 35–37] showed no significant difference between SG-TB and SG (OR = 1.25, 95% CI 0.53–2.91, *p* = 0.61), while four studies (*n* = 243) [27, 28, 33, 34] revealed equivalent remission rates between SG-TB and RYGB (OR = 0.85, 95% CI 0.38–1.87, *p* = 0.68), both with minimal heterogeneity (I^2^ = 0%) (Supplemental Fig. 1A and B)

Dyslipidemia analysis of four studies (*n* = 289) [26, 30, 35, 37] indicated a non-significant trend favoring SG-TB over SG (OR = 2.18, 95% CI 0.92–5.17, *p* = 0.07) with homogeneous results across studies (I^2^ = 0%). Meta-analysis of four studies (*N* = 86) [26, 30, 35, 37] revealed that SG-TB demonstrates significantly higher GERD remission rates compared to SG (OR = 6.90, 95% CI 1.98–24.09, *p* = 0.002) with no heterogeneity (I^2^ = 0%). (Table 3) (Supplemental Fig. 1C and D)

### Surgical parameters and nutritional outcomes

Analysis of perioperative complications revealed comparable safety profiles across procedures. Six studies (*n* = 528) [26, 29, 35, 36, 38] showed no significant difference between SG-TB and SG (OR = 1.03, 95% CI0.34–3.07, *p* = 0.96) with moderate heterogeneity (I^2^ = 44%). Similarly, four studies (*n* = 392) [27, 28, 33, 34] demonstrated equivalent complication rates between SG-TB and RYGB (OR = 1.32, 95% CI0.62–2.78, *p* = 0.47) with minimal heterogeneity (I^2^ = 0%) (Table 3) (Fig. 3C and D).

The comparative analysis of nutritional parameters, as detailed in Supplemental Table 6, was limited by inconsistent reporting across studies and predominantly short-term follow-up (mainly 12 months). In the comparison between SG-TB and SG, albumin and hemoglobin levels appeared largely comparable where reported. However, SG demonstrated more favorable postoperative calcium and vitamin D levels in the single study reporting these outcomes. Conversely, SG-TB showed a potential advantage in maintaining serum iron and vitamin B12 levels, though these findings were inconsistent across the limited datasets.

When compared to RYGB, SG-TB demonstrated a trend toward better preservation of iron status and vitamin B12 levels. Vitamin D levels also appeared higher with SG-TB in the single reporting study. The incidence of hypoalbuminemia and anemia was low in both groups were reported. The limited reporting and short follow-up duration preclude definitive conclusions regarding long-term nutritional safety.

Analysis of baseline glycemic parameters revealed significantly higher preoperative HbA1c levels in SG-TB patients compared to SG controls (MD = 0.53%, 95% CI 0.12–0.93%, *p* = 0.01) across four studies. Operative time analysis demonstrated significantly longer procedures for SG-TB versus SG (MD = 30.4 min, 95% CI 8.6–52.5, *p* = 0.006), indicating greater technical complexity. Complete baseline metabolic profiles and surgical details are available in Supplemental Table 7.

## Discussion

Our systematic review and meta-analysis elucidate the comparative efficacy and safety profile of SG-TB against the established benchmarks of SG and RYGB. The synthesized evidence positions SG-TB as a technically simplified yet metabolically potent procedure, demonstrating superior weight loss over SG, non-inferiority to RYGB, and a consistently favorable safety profile.

The significantly greater weight loss, both in terms of TWL and EWL, observed with SG-TB compared to SG underscores the limitation of a purely restrictive approach, particularly in higher BMI populations [11, 14]. While SG relies primarily on gastric volume reduction and ghrelin suppression [50, 51], SG-TB incorporates a crucial enteric component. The creation of a gastro-enteric anastomosis, typically 250–300 cm from the ileocecal valve, orchestrates a more profound metabolic response by altering nutrient transit [17, 19]. The mechanisms for this enhanced efficacy are multifactorial. First, the expedited delivery of undigested nutrients to the distal small intestine potently stimulates the release of L-cell hormones, most notably glucagon-like peptide-1 (GLP-1) and peptide YY (PYY) [18, 52]. GLP-1 enhances glucose-dependent insulin secretion, promotes satiety, and inhibits gastric emptying, while PYY acts as a potent anorexigenic signal in the central nervous system [20, 53, 54]. This “ileal brake” phenomenon is engaged more robustly in SG-TB than in SG, contributing to reduced appetite and improved glycemic control. Second, the bipartition of food stream allows a portion of nutrients to follow the physiological path, potentially mitigating the risk of severe malabsorption and nutritional deficiencies compared to classic biliopancreatic diversions [16, 55]. When compared to RYGB, the weight loss equivalence suggests that the metabolic benefits of a single, distal anastomosis may parallel those achieved by the more complex Roux-en-Y configuration, albeit through a different anatomical and physiological arrangement.

The substantial heterogeneity observed in T2DM remission analysis between SG-TB and SG (I^2^ = 87%) likely reflects multiple factors: significant baseline differences in diabetic severity (with SG-TB patients having longer diabetes duration, higher HbA1c, and greater insulin use), variations in surgical technique including common channel length, and differing definitions of diabetes remission across studies (Supplemental Table 5). The pronounced trend towards superior T2DM remission with SG-TB over SG, even in the face of less favorable preoperative glycemic status (higher baseline HbA1c), highlights its inherent metabolic advantage. This effect is only partially explained by greater weight loss. The foregut and hindgut hypotheses, initially proposed for RYGB, are highly relevant here [56, 57]. The hindgut hypothesis is strongly supported by our findings. The rapid nutrient exposure to the ileum, as discussed, enhances GLP-1 secretion. The potent incretin effect of GLP-1 is a key driver of postprandial insulin secretion and beta-cell function improvement, often leading to diabetes remission independent of weight loss [58–60]. Concurrently, the foregut hypothesis posits that bypassing the duodenum and proximal jejunum excludes a putative “anti-incretin” signal, potentially involving the downregulation of unfavorable hormonal pathways. SG-TB achieves both: it diverts nutrients away from the foregut while ensuring their rapid delivery to the hindgut. This dual mechanism may explain why its metabolic efficacy rivals that of RYGB, as evidenced by the comparable remission rates between the two procedures [27, 28, 33, 34]. Furthermore, the favorable, though not always statistically significant, trends in dyslipidemia remission with SG-TB are likely mediated by similar pathways. Enhanced GLP-1 signaling has been shown to improve lipid metabolism, and the altered biliary flow and nutrient absorption may directly reduce cholesterol and triglyceride levels [61–63].

The comparable rates of perioperative complications between SG-TB and both SG and RYGB are a cornerstone of its clinical viability. Although SG-TB requires a longer operative time than SG, reflecting the added complexity of the intestinal anastomosis, this does not translate into a higher risk profile. This is a critical finding for surgeons considering adopting these techniques. The significantly higher GERD remission rates observed with SG-TB compared to SG are particularly noteworthy given theoretical concerns about bile reflux [64, 65]. This finding may be attributed to more effective weight reduction and potential alterations in gastroesophageal dynamics. However, the variability in GERD assessment methods across studies necessitates cautious interpretation, and future research should employ standardized objective measures to validate these findings.

Based on the systematic assessment of studies not included in the quantitative synthesis, several relevant comparisons between different technical modifications of SG-TB emerge. Notably, the SASI bypass, its jejunal variant (SAS-J), and the Braun-anastomosis modified SASI (SASI-B) represent important evolutionary technical adaptations aimed at optimizing the balance between metabolic efficacy, weight loss, and safety profile. Collective analysis of the studies by Demir, Topart, and Arslan [44, 48, 49] showed that SG-TB and SASI demonstrate substantial efficacy in weight loss and metabolic control. These procedures consistently achieve significant weight reduction and high rates of type 2 diabetes remission. The technical simplification offered by the loop design (SASI) further results in a notably shorter operative time, enhancing procedural efficiency. Importantly, when compared to malabsorptive techniques like BPD-DS, SG-TB exhibits a superior safety profile in superobese patients, markedly reducing the risks of protein malnutrition and severe diarrheal complications. The prospective randomized trial by Sewefy et al. [43] provides high-quality evidence directly comparing SASI and SAS-J bypass. While SASI demonstrated superior excess weight loss (%EWL 94.8 vs. 90.6%), this came at the cost of significantly higher overall nutritional deficiencies (58 vs. 27.6%) and higher reoperation rates (11.4 vs. 2.3%). This underscores a critical trade-off: the more distal intestinal bypass in SASI enhances weight loss but increases malabsorptive risks. In contrast, SAS-J, with its shorter biliopancreatic limb, preserves strong weight loss efficacy while substantially improving the nutritional safety profile. Furthermore, the comparative study by Hosseini et al. [46] were similar to the findings of Sewefy et al. [43], demonstrating that SAS-J bypass was associated with a significantly lower reversal surgery rate compared to SASI (0.0 vs. 5.5%), directly attributable to fewer instances of excessive weight loss and malnutrition. This consolidates the premise that a shorter biliopancreatic limb, as in SAS-J, offers a more favorable risk-benefit ratio for most patients. Concurrently, technical modifications to address bile reflux in SASI were evaluated. Hosseini et al. [45] and Widjaja et al. [47] investigated the addition of a Braun entero-enterostomy to the SASI loop (SASI-B or B-TB). Both pilot studies reported a consistent trend—though not always statistically significant due to sample size—towards reduced objective bile reflux and related symptoms like esophagitis. Additionally, based on recent animal model evidence, SG-TB with Braun anastomosis (BTB) demonstrates comparable metabolic and weight-loss outcomes to RYTB, while notably reducing biliary reflux, suggesting a potentially superior safety profile for selected patients [66]. This suggests Braun anastomosis is a promising technical refinement to mitigate one of the theoretical drawbacks of single-anastomosis procedures without converting to a more complex Roux-en-Y reconstruction.

Based on evidence from animal and mechanistic studies, SG-TB demonstrates distinct metabolic advantages. In rodent models, SG-TB provides superior and better-maintained weight loss compared to RYGB over the long term, accompanied by enhanced insulin secretion without compromising nutritional status [67, 68]. Further investigations indicate that SG-TB and RYGB induce comparable post-operative elevations in gut hormones such as GLP-1 and PYY; however, SG-TB may achieve superior glycemic control through a more potent insulin secretory response [69]. The level of β-cell function after surgery is a strong predictor of hyperglycemia resolution, and a disconnect exists between postprandial GLP-1 levels and β-cell function among different procedures [69]. Anatomically, SG-TB preserves partial nutrient transit through the duodenum, a feature hypothesized to mitigate severe nutritional deficiencies and help maintain physiological regulation of metabolic pathways. This is supported by findings that SASI (a variant similar to SG-TB) presents a lower risk of hypoalbuminemia and iron deficiency compared to the more malabsorptive SADI-S [70]. Furthermore, significant increases in testosterone levels in males with obesity and T2DM after SG-TB correlate strongly with BMI loss, highlighting its systemic metabolic benefits [71]. Collectively, these preclinical data position SG-TB as a procedure combining potent metabolic efficacy with a favorable safety profile, mediated through balanced hormonal regulation and nutrient absorption, meriting further validation in future clinical studies.

In summary, the evolution of SG-TB illustrates a clear trajectory: from the effective but higher-risk SASI towards modified procedures that prioritize safety—either by shifting the anastomosis proximally (SAS-J) or by incorporating a bile-diverting Braun anastomosis (SASI-B). These adaptations successfully maintain the compelling metabolic outcomes characteristic of the bipartition principle while significantly mitigating its key nutritional and reflux-related complications. Future research should focus on long-term outcomes and standardized techniques to solidify the place of these promising procedures in the bariatric arsenal.

### Limitations and future direction

Our findings must be interpreted within the context of the available evidence. The predominance of retrospective studies introduces potential selection bias, and the technical heterogeneity in anastomotic location and common channel length across studies contributes to clinical and statistical heterogeneity. The lack of long-term data, particularly on nutritional outcomes and weight recurrence, remains a significant gap.

While our analysis of available nutritional data suggests generally comparable outcomes between SG-TB and established procedures in the short-term, the malabsorptive component of SG-TB warrants careful consideration of potential long-term nutritional consequences. The limited data available, predominantly with 12-month follow-up, reveals a complex nutritional profile: SG-TB appears to demonstrate better preservation of iron and vitamin B12 levels compared to RYGB, while SG may be associated with more favorable calcium and vitamin D levels than SG-TB. These findings align with recent literature highlighting the importance of vigilant nutritional monitoring after malabsorptive procedures [72–74]. The preservation of partial nutrient transit through the duodenum in SG-TB may offer some protection against severe deficiencies compared to purely malabsorptive techniques, but long-term data remain essential to fully characterize the nutritional safety profile of these procedures.

However, while our analysis demonstrates comparable short-term safety profiles, the predominance of 12-month follow-up data (11 of 13 studies) limits assessment of long-term nutritional sequelae characteristic of malabsorptive procedures. The potential for late-emerging deficiencies necessitates cautious interpretation of our safety findings and underscores the need for long-term nutritional surveillance after SG-TB procedures.

In addition, it is important to recognize that ‘SG-TB’ serves as an umbrella term encompassing several distinct procedures with varying technical details, particularly regarding anastomotic location and common channel length (Supplemental Table 8). This methodological heterogeneity, while necessitated by the available literature, may influence outcomes and should be considered when interpreting our findings. The evolving technical modifications—such as the shift from SASI to SAS-J to reduce nutritional deficiencies—highlight the importance of procedure-specific evaluation rather than considering SG-TB as a monolithic entity.

Furthermore, it should be noted that many SG-TB variants are still considered innovative procedures within the metabolic surgery landscape. Current guidelines from major societies recommend that such procedures be performed within structured research protocols with appropriate ethical oversight and long-term follow-up [75]. Our findings support the continued investigation of SG-TB procedures but should be interpreted within this context of responsible surgical innovation.

Future research must prioritize prospective, randomized trials with standardized surgical protocols and long-term follow-up. Investigations should move beyond efficacy to elucidate the precise physiological mechanisms, including detailed hormonal profiling, bile acid dynamics, and microbiome changes following SG-TB compared to SG and RYGB [76–79]. Understanding these mechanisms will allow for better patient selection and procedure personalization.

## Conclusions

SG-TB procedures represent a significant evolution in metabolic-bariatric surgery, effectively blending the principles of restriction and metabolic modulation through a simplified anatomical construct. They offer a compelling alternative, providing superior weight loss to SG and matching the metabolic efficacy of RYGB for T2DM and weight loss, all while maintaining a comparable short-term safety footprint. However, these findings must be interpreted with caution given the substantial heterogeneity in T2DM outcomes, the investigational status of many SG-TB variants, and the critical limitation of predominantly short-term follow-up data. The procedural heterogeneity under the SG-TB umbrella and potential long-term nutritional implications highlighted in our analysis warrant careful consideration. While our results position certain SG-TB variants as promising surgical options for severe obesity, they should currently be considered within the context of ongoing surgical innovation rather than as established alternatives to conventional procedures. Future studies with standardized techniques, longer follow-up, and comprehensive nutritional assessment are essential to definitively establish the risk-benefit profile of these procedures.

## Supplementary Information

Below is the link to the electronic supplementary material.

Supplementary Material 1: Figure S1. Forest plots of hypertension, dyslipidemia and GERD remission: SG-TB vs. SG and RYGB.**A** Odds ratios (OR) for hypertension remission comparing SG-TB with SG. **B** Odds ratios for hypertension remission comparing SG-TB with RYGB. **C** Odds ratios for dyslipidemia remission comparing SG-TB with SG. **D** Odds ratios for GERD remission comparing SG-TB with SG.

Supplementary Material 2

## Data Availability

The database used and/or analyzed during the current study is not publicly available (to maintain privacy) but can be available from the corresponding author on reasonable request.
