# Supplementary figures and images for: Efficacy and safety of single-anastomosis gastric bypass variants versus sleeve gastrectomy or Roux-en-Y gastric bypass: a systematic review and meta-analysis
Source: Updates Surg. 2026 Jan 28;78(2):893–907. doi: 10.1007/s13304-025-02518-1 (PMC13212818; doi:10.1007/s13304-025-02518-1)

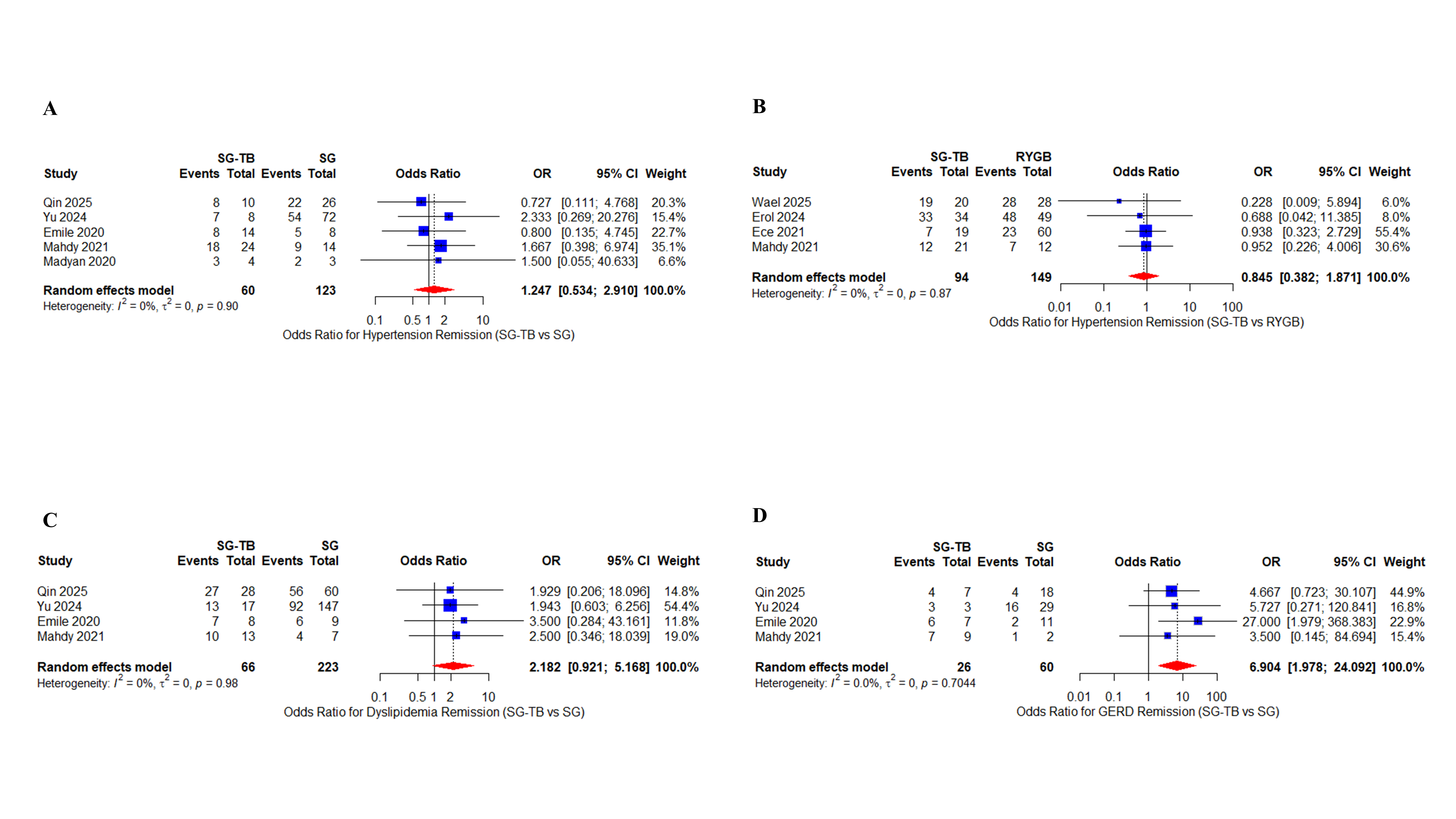

Supplement: Supplementary file 1 — Supplementary Material 1: Figure S1. Forest plots of hypertension, dyslipidemia and GERD remission: SG-TB vs. SG and RYGB.A Odds ratios (OR) for hypertension remission comparing SG-TB with SG. B Odds ratios for hypertension remission comparing SG-TB with RYGB. C Odds ratios for dyslipidemia remission comparing SG-TB with SG. D Odds ratios for GERD remission comparing SG-TB with SG. [file 13304_2025_2518_MOESM1_ESM.tif]
