# Supplementary material for: Efficacy and safety of single-anastomosis gastric bypass variants versus sleeve gastrectomy or Roux-en-Y gastric bypass: a systematic review and meta-analysis
Source: Updates Surg. 2026 Jan 28;78(2):893–907. doi: 10.1007/s13304-025-02518-1 (PMC13212818; doi:10.1007/s13304-025-02518-1)
Supplement: Supplementary file 2 — Supplementary Material 2 [file 13304_2025_2518_MOESM2_ESM.docx]

Supplemental Table 1. Characteristics of relevant studies included in systematic review but not in meta-analysis

| Author | Year | Country | Study design | Patients n | Age (mean, yrs) | BMI (mean, kg/m2) | Follow-up, m | Outcomes |
| --- | --- | --- | --- | --- | --- | --- | --- | --- |
| Sewefy ^[43]^ | 2025 | Egypt | RCT | 90 SASI  90 SASJ | 36.1  35.5 | 47.7±7.2  47.5±6 | 12 | Weight Comorbidities  Complication |
| Demir ^[44]^ | 2023 | Turkey | Retrospective | 32, SASI, T2DM  15, SG-TB, T2DM | 48.8  51.2 | 38.2±4.3  41.0±5.1 | 12 | Weight Comorbidities |
| Hosseini ^[45]^ | 2023 | Iran | Retrospective | 16, SASI  12, SASI-Braun | 40.8  38.2 | 43.5±3.0  44.8±4.3 | 12 | Weight Comorbidities |
| Hosseini ^[46]^ | 2022 | Iran | Retrospective | 138 SASI  24 SASJ | 41.5  43.4 | 45.4±4.6  45.2±3.8 | 12 | Weight  Comorbidities |
| Widjaja ^[47]^ | 2021 | China | Retrospective | 40, SG-TB  10, SASI-Braun | 34.5  37.6 | 37.2±5.8  39.1±4.4 | 3 | Weight  Complication |
| Topart ^[48]^ | 2019 | France | Retrospective | 71, SG-TB  71, BPD-DS | 40  42 | 51.6±5  53.7±5.2 | 12 | Weight  Complication  Comorbidities |
| Arslan ^[49]^ | 2019 | Turkey | Prospective | 7, SG-TB, T2DM  15, SASI, T2DM | 50  53.4 | 32.6±1.7  35.1 ± 4.2 | 3 | Weight  Complication  Comorbidities |

RCT: randomized clinical trial; BMI, body mass index; T2DM, type 2 diabetes mellitus; SASI, anastomosis sleeve ileal bypass; SASJ, single anastomosis sleeve jejunal bypass, SASI-B, SASI with Braun anastomosis; SG-TB, SG with transit bipartition, RYGB, Roux-en-Y gastric bypass; SG, sleeve gastrectomy; BPD-DS, Biliopancreatic Diversion-Duodenal Switch

Supplemental Table 2. Summary of findings of relevant studies included in systematic review but not in meta-analysis

| Author (ref) | Study design | Follow-up | 1 year  %EWL | %TWL | Resolved %  T2DM | Hypertension | Dyslipidemia | Perioperative  Complication | Operation time |
| --- | --- | --- | --- | --- | --- | --- | --- | --- | --- |
| Sewefy ^[43]^ | SASI  SASJ | 1 year | 94.8  90.6 | 44.4  42.5 | 95%  91% | 83.3%  81.3% | 83.3%  78.3% | 53.4%  34.5% | NR  NR |
| Demir ^[44]^ | SASI  SG-TB | 1 year | 79.3  61.8 | NR  NR | 68.8%  73.3% | NR  NR | NR  NR | NR  NR | 183.8±21.9  224.3±14.5 |
| Hosseini ^[45]^ | SASI  SASI-B | 1 year | NR  NR | 44.9  37.8 | NR  NR | NR  NR | NR  NR | NR  NR | NR  NR |
| Hosseini ^[46]^ | SASI  SASJ | 1 year | 87.9  76.8 | 38.7  33.8 | 84.6%  83.3% | 79.3%  50% | 76.9%  70% | 10.1%  8.2% | NR  NR |
| Topart ^[48]^ | SG-TB  BPDDS | 1 year | 78.6  83.7 | 41.3  45 | 90%  88% | 81.8%  61.9% | NR  NR | 4.2%  5.6% | NR  NR |
| Arslan ^[49]^ | SG-TB  SASI | 3 month | NR  NR | NR  NR | 85.7%  66.7% | NR  NR | NR  NR | 0%  0% | 231 ± 27.3  192.8 ± 29.7 |

T2DM, type 2 diabetes mellitus; SASI, anastomosis sleeve ileal bypass; SASJ, single anastomosis sleeve jejunal bypass, SASI-B, SASI with Braun anastomosis; SG-TB, SG with transit bipartition, RYGB, Roux-en-Y gastric bypass; SG, sleeve gastrectomy; BPD-DS, Biliopancreatic Diversion-Duodenal Switch; EWL, excess weight loss; TWL, total weight loss; NR, not reported.

Supplemental Table 3. Detail summary of weight loss of SG-TB surgery compared with SG or RYGB of all included studies

| Author (ref) | Study design | 3 month  %EWL | %TWL | 6 month  %EWL | %TWL | 1 year  %EWL | %TWL | 2 years  %EWL | %TWL | 3 years  %EWL | %TWL | 4 years  %EWL | %TWL | 5 years  %EWL | %TWL |
| --- | --- | --- | --- | --- | --- | --- | --- | --- | --- | --- | --- | --- | --- | --- | --- |
| Qin ^[30]^ | SASI  SG | NR  NR | NR  NR | NR  NR | NR  NR | NR  NR | 37  29.9 | NR  NR | NR  NR | NR  NR | NR  NR | NR  NR | NR  NR | NR  NR | NR  NR |
| Wael ^[31]^ | SASI  RYGB | 11.7  7.2 | 7.1  4.8 | 25.6  17.1 | 15.5  10.4 | 33.7  29.3 | 20.4  17.7 | NR  NR | NR  NR | NR  NR | NR  NR | NR  NR | NR  NR | NR  NR | NR  NR |
| Sewefy ^[43]^ | SASI  SASJ | 38.7  37.1 | 17.9  17 | 62.4  58.9 | 29.1  27.2 | 94.8  90.6 | 44.4  42.5 | NR  NR | NR  NR | NR  NR | NR  NR | NR  NR | NR  NR | NR  NR | NR  NR |
| Erol ^[32]^ | SASI  RYGB | NR  NR | NR  NR | 50  38.4 | 21.3  15.4 | 96.7  90 | 39  36.9 | 97.7  93.6 | 41.1  41.6 | NR  NR | NR  NR | NR  NR | NR  NR | NR  NR | NR  NR |
| Foschi ^[33]^ | SASI  SG  SADI-S | NR  NR  NR | NR  NR  NR | 65  52  60 | NR  NR  NR | 77.5  65  76 | NR  NR  NR | NR  NR  NR | NR  NR  NR | 69  68  56 | NR  NR  NR | NR  NR  NR | NR  NR  NR | 58  45  58 | NR  NR  NR |
| Yu ^[34]^ | SASI  SG  OAGB | NR  NR  NR | NR  NR  NR | 63  67.5  56.6 | 17.8  19.5  21.5 | 88.2  74.9  84.1 | 27.7  26.5  25.8 | NR  NR  NR | NR  NR  NR | NR  NR  NR | NR  NR  NR | NR  NR  NR | NR  NR  NR | NR  NR  NR | NR  NR  NR |
| Demir ^[44]^ | SASI  SG-TB | NR  NR | NR  NR | NR  NR | NR  NR | 79.3  61.8 | NR  NR | NR  NR | NR  NR | NR  NR | NR  NR | NR  NR | NR  NR | NR  NR | NR  NR |
| Hosseini ^[45]^ | SASI  SASI-B | NR  NR | NR  NR | NR  NR | NR  NR | NR  NR | 44.9  37.8 | NR  NR | NR  NR | NR  NR | NR  NR | NR  NR | NR  NR | NR  NR | NR  NR |
| Yildirak ^[35]^ | SASI  SG | NR  NR | NR  NR | 55.2  57.4 | 24.5  26.3 | 71.7  69.7 | 32.4  31.9 | NR  NR | NR  NR | NR  NR | NR  NR | NR  NR | NR  NR | NR  NR | NR  NR |
| Kirkil ^[36]^ | SG-TB  SG  OAGB | NR  NR  NR | NR  NR  NR | NR  NR  NR | NR  NR  NR | 98.3  89  84.7 | NR  NR  NR | NR  NR  NR | NR  NR  NR | NR  NR  NR | NR  NR  NR | NR  NR  NR | NR  NR  NR | NR  NR  NR | NR  NR  NR |
| Hosseini ^[46]^ | SASI  SASJ | NR  NR | NR  NR | NR  NR | NR  NR | 87.9  76.8 | 38.7  33.8 | NR  NR | NR  NR | NR  NR | NR  NR | NR  NR | NR  NR | NR  NR | NR  NR |
| Ece ^[37]^ | SG-TB  RYGB | 28.4  21.6 | 9.4  12.1 | 42.2  45.8 | 18.6  19.8 | 29.7  30.9 | 68.4  71.3 | NR  NR | NR  NR | NR  NR | NR  NR | NR  NR | NR  NR | NR  NR | NR  NR |
| Mahdy ^[38]^ | SASI  RYGB | NR  NR | NR  NR | NR  NR | NR  NR | 78.5  79.4 | 30.4  33.4 | NR  NR | NR  NR | NR  NR | NR  NR | NR  NR | NR  NR | NR  NR | NR  NR |
| Mahdy ^[39]^ | SASI  SG  OAGB | NR  NR  NR | NR  NR  NR | 80.2  57.9  50.8 | 27.7  26.2  24 | 87.6  72.5  65.9 | 36.1  31.6  29.1 | NR  NR  NR | NR  NR  NR | NR  NR  NR | NR  NR  NR | NR  NR  NR | NR  NR  NR | NR  NR  NR | NR  NR  NR |
| Widjaja ^[47]^ | SG-TB  SASI-B | 22.2  19.7 | NR  NR | NR  NR | NR  NR | NR  NR | NR  NR | NR  NR | NR  NR | NR  NR | NR  NR | NR  NR | NR  NR | NR  NR | NR  NR |
| Madyan ^[40]^ | SASI  SG | NR  NR | NR  NR | 44.3  40.3 | NR  NR | 65.2  57.4 | NR  NR | NR  NR | NR  NR | NR  NR | NR  NR | NR  NR | NR  NR | NR  NR | NR  NR |
| Emile ^[41]^ | SASI  SG | NR  NR | NR  NR | 46.2  43.4 | 25.7  24.8 | 72.6  60.4 | 37.8  33.3 | NR  NR | NR  NR | NR  NR | NR  NR | NR  NR | NR  NR | NR  NR | NR  NR |
| Topart ^[48]^ | SG-TB  BPDDS | NR  NR | NR  NR | NR  NR | NR  NR | 78.6  83.7 | 41.3  45 | NR  NR | NR  NR | NR  NR | NR  NR | NR  NR | NR  NR | NR  NR | NR  NR |
| Arslan ^[49]^ | SG-TB  SASI | 75.2  62.2 | NR  NR | NR  NR | NR  NR | NR  NR | NR  NR | NR  NR | NR  NR | NR  NR | NR  NR | NR  NR | NR  NR | NR  NR | NR  NR |
| Yormaz ^[42]^ | SG-TB  SG  BPDDS | NR  NR  NR | NR  NR  NR | NR  NR  NR | NR  NR  NR | NR  NR  NR | 27.1  23.2  22.4 | NR  NR  NR | NR  NR  NR | NR  NR  NR | NR  NR  NR | NR  NR  NR | NR  NR  NR | NR  NR  NR | NR  NR  NR |

SASI, anastomosis sleeve ileal bypass; SASJ, single anastomosis sleeve jejunal bypass, SASI-B, SASI with Braun anastomosis; SG-TB, SG with transit bipartition, RYGB, Roux-en-Y gastric bypass; SG, sleeve gastrectomy; BPD-DS, Biliopancreatic Diversion-Duodenal Switch; EWL, excess weight loss; TWL, total weight loss; NR, not reported.

Supplemental Table 4. Detail summary of comorbidities remission of SG-TB surgery compared with SG or RYGB

| Author (ref) | Study design | Follow-up | Comorbidities  HbA1C | HOMA-IR | Resolved %  T2DM | Cholesterol | Triglycerides | LDL | Resolved %  Dyslipidemia | Resolved %  Hypertension | Uric acid | Resolved %  GERD | Resolved %  OSAS |
| --- | --- | --- | --- | --- | --- | --- | --- | --- | --- | --- | --- | --- | --- |
| Qin ^[30]^ | SASI  SG | 1 year | 5.4±1.1  5.2±1.2 | NR  NR | 90%  50% | 4.8±0.6  5.3±0.8 | 1.0±0.2  1.4±0.5 | 2.9±0.5  3.4±0.8 | 96.4%  93.3% | 80%  84.6% | 263.1±51.5  338.5±52.6 | 57.1%  22.2% | NR  NR |
| Wael ^[31]^ | SASI  RYGB | 1 year | NR  NR | NR  NR | 88.9%  87.5% | NR  NR | NR  NR | NR  NR | 100%  100% | 95%  100% | NR  NR | 84.6%  100% | 86.7%  91.7% |
| Sewefy ^[43]^ | SASI  SASJ | 1 year | NR  NR | NR  NR | 95%  91% | NR  NR | NR  NR | NR  NR | 83.3%  78.3% | 83.3%  81.3% | NR  NR | 84.6%  81.3% | 86.6%  84.2% |
| Erol ^[32]^ | SASI  RYGB | 2 year | 5.0±0.5  5.0±0.6 | NR  NR | 96.1%  95.6% | NR  NR | NR  NR | NR  NR | 100%  100% | 97.1%  98% | NR  NR | NR  NR | NR  NR |
| Foschi ^[33]^ | SASI  SG  SADI-S | 5 year | 5.2±0.8  6.6±0.9  5.2±0.8 | NR  NR  NR | 80%  30%  80% | NR  NR  NR | NR  NR  NR | NR  NR  NR | NR  NR  NR | NR  NR  NR | NR  NR  NR | NR  NR  NR | NR  NR  NR |
| Yu ^[34]^ | SASI  SG  OAGB | 1 year | NR  NR  NR | 2.6±0.5  3.0±0.8  2.5±0.7 | 83.3%  63.9%  94.7% | NR  NR  NR | 1.2±0.3  1.3±0.2  1.4±0.4 | NR  NR  NR | 76.5%  62.6%  77.3% | 87.5%  75%  84.2% | NR  NR  NR | 100%  55.2%  100% | 100%  93.8%  92.3% |
| Demir ^[44]^ | SASI  SG-TB | 1 year | 6.5±0.7  6.6±0.7 | NR  NR | 68.8%  73.3% | NR  NR | NR  NR | NR  NR | NR  NR | NR  NR | NR  NR | NR  NR | NR  NR |
| Hosseini ^[45]^ | SASI  SASI-B | 1 year | NR  NR | NR  NR | NR  NR | NR  NR | NR  NR | NR  NR | NR  NR | NR  NR | NR  NR | NR  NR | NR  NR |
| Yildirak ^[35]^ | SASI  SG | 1 year | 6.3±1.0  6.1±1.0 | NR  NR | 32.3%  46.7% | NR  NR | NR  NR | NR  NR | NR  NR | NR  NR | NR  NR | NR  NR | NR  NR |
| Kirkil ^[36]^ | SG-TB  SG  OAGB | 1 year | 5.6±1.3  5.2±1.1  5.5±1.5 | NR  NR  NR | 76.6%  95.3%  84.6% | NR  NR  NR | NR  NR  NR | NR  NR  NR | NR  NR  NR | NR  NR  NR | NR  NR  NR | NR  NR  NR | NR  NR  NR |
| Hosseini ^[46]^ | SASI  SASJ | 1 year | NR  NR | NR  NR | 84.6%  83.3% | 3.4±1.1  3.5±0.7 | 1.2±0.6  1.3±0.4 | 2.3±1.0  2.5±0.6 | 76.9%  70% | 79.3%  50% | NR  NR | NR  NR | 87.9%  87.5% |
| Ece ^[37]^ | SG-TB  RYGB | 1 year | 5.4±1.1  5.5±1.0 | 2.2±0.9  2.1±1.2 | 77%  79.6% | NR  NR | NR  NR | NR  NR | NR  NR | 36.8%  38.3% | NR  NR | NR  NR | NR  NR |
| Mahdy ^[38]^ | SASI  RYGB | 1 year | 5.8±2.7  5.4±1.5 | NR  NR | 82.7%  73.7% | NR  NR | NR  NR | NR  NR | 76.9%  100% | 57.1%  58.3% | NR  NR | NR  NR | 20%  0% |
| Mahdy ^[39]^ | SASI  SG  OAGB | 1 year | NR  NR  NR | NR  NR  NR | 97.7%  71.4%  85.7% | NR  NR  NR | NR  NR  NR | NR  NR  NR | 76.9%  57.1%  78.5% | 75%  64.3%  84% | NR  NR  NR | 77.7%  50%  62.9% | 100%  100%  100% |
| Widjaja ^[47]^ | SG-TB  SASI-B | 3 month | NR  NR | NR  NR | NR  NR | NR  NR | NR  NR | NR  NR | NR  NR | NR  NR | NR  NR | NR  NR | NR  NR |
| Madyan ^[40]^ | SASI  SG | 1 year | NR  NR | NR  NR | 100%  100% | NR  NR | NR  NR | NR  NR | NR  NR | 75%  66.7% | NR  NR | NR  NR | NR  NR |
| Emile ^[41]^ | SASI  SG | 1 year | NR  NR | NR  NR | 95.8%  70% | NR  NR | NR  NR | NR  NR | 87.5%  66.7% | 57.1%  62.5% | NR  NR | 85.7%  18.2% | NR  NR |
| Topart ^[48]^ | SG-TB  BPDDS | 1 year | NR  NR | NR  NR | 90%  88% | NR  NR | NR  NR | NR  NR | NR  NR | 81.8%  61.9% | NR  NR | NR  NR | 84%  78% |
| Arslan ^[49]^ | SG-TB  SASI | 3 month | 6.0±0.4  6.6±0.7 | NR  NR | 85.7%  66.7% | NR  NR | NR  NR | NR  NR | NR  NR | NR  NR | NR  NR | NR  NR | NR  NR |
| Yormaz ^[42]^ | SG-TB  SG  BPDDS | 1 year | 6.0±1.5  6.8±1.4  6.2±1.1 | 1.7±0.4  2.1±0.6  2.3±0.3 | 82.9%  35.3%  91.4% | 4.5±0.9  4.8±0.5  4.5±0.8 | NR  NR  NR | NR  NR  NR | NR  NR  NR | NR  NR  NR | NR  NR  NR | NR  NR  NR | NR  NR  NR |

HbA1C, Glycated Haemoglobin A1c; LDL, low density lipoprotein, GERD, Gastroesophageal Reflux Disease; OSAS, Obstructive Sleep Apnea Syndrome; HOMA-IR, Homeostatic Model Assessment of Insulin Resistance; T2DM, type 2 diabetes mellitus; SASI, anastomosis sleeve ileal bypass; SASJ, single anastomosis sleeve jejunal bypass, SASI-B, SASI with Braun anastomosis; SG-TB, SG with transit bipartition, RYGB, Roux-en-Y gastric bypass; SG, sleeve gastrectomy; BPD-DS, Biliopancreatic Diversion-Duodenal Switch; NR, not reported.

Supplemental Table 5. Summary of T2DM characteristics of studies included in the meta-analysis

| Author (ref) | Study design | Duration of T2DM, years | FPG  mmol/L | HbA1C  % | C-pep  ng/mL | Insulin-use | FPG  mmol/L | HbA1C  % | C-pep  ng/mL | HOMA-IR | Resolved  T2DM% | Definition of remission |
| --- | --- | --- | --- | --- | --- | --- | --- | --- | --- | --- | --- | --- |
| **SG-TB vs. SG** | | **Baseline** |  |  |  |  | **Postoperative** | |  |  |  |  |
| Qin ^[30]^ | SASI  SG | NR  NR | 7.4±4.3  6.7±3.3 | 6.9±1.9  6.2±1.2 | 3.8±1.0  2.6±0.7 | NR  NR | 5.1±1.1  5.3±1.2 | 5.4±1.1  5.2±1.2 | 1.2±0.6  1.9±0.8 | NR  NR | 90%  50% | A1C <6.5% without anti-diabetes medications. |
| Yu ^[34]^ | SASI  SG | NR  NR | 7.1±2.9  6.0±2.3 | 6.9±2.2  6.1±1.2 | 3.5±1.4  4.2±1.6 | NR  NR | 4.8±0.7  5.0±1.2 | NR  NR | NR  NR | 2.6±0.5  3.0±0.8 | 83.3%  63.9% | FPG<100 mg/dL without anti-diabetes medications |
| Yildirak ^[35]^ | SASI  SG | 10  6 | 11.6±3.7  9.5±3.4 | 9.4±1.5  8.6±2.1 | NR  NR | 96.8%  53.3% | 6.2±1.7  6.0±2.0 | 6.3±1.0  6.1±1.0 | NR  NR | NR  NR | 32.3%  46.7% | FPG<100 mg/dL or A1C <6% without anti-diabetes medications. |
| Kirkil ^[36]^ | SG-TB  SG | 9.0  2 | NR  NR | 9.1  6.7 | 2.9  4.6 | 76.6%  5.2% | NR  NR | 5.6±1.3  5.2±1.1 | NR  NR | NR  NR | 76.6%  95.3% | FPG<100 mg/dL or A1C <6.5% without anti-diabetes medications. |
| Mahdy ^[39]^ | SASI  SG | NR  NR | NR  NR | NR  NR | NR  NR | NR  NR | NR  NR | NR  NR | NR  NR | NR  NR | 97.7%  71.4% | FPG<110 mg/dL or A1C <6.5% without anti-diabetes medications. |
| Madyan ^[40]^ | SASI  SG | NR  NR | NR  NR | NR  NR | NR  NR | NR  NR | NR  NR | NR  NR | NR  NR | NR  NR | 100%  100% | FPG<110 mg/dL or A1C <6.5% without anti-diabetes medications. |
| Emile ^[41]^ | SASI  SG | NR  NR | NR  NR | NR  NR | NR  NR | NR  NR | NR  NR | NR  NR | NR  NR | NR  NR | 95.8%  70% | FPG<100 mg/dL or A1C <6% without anti-diabetes medications. |
| Yormaz ^[42]^ | SG-TB  SG | 15.4±8.5  13.3±5.3 | 12.9±0.7  13.1±0.8 | 9.0±1.2  9.0±1.2 | 2.4±1.4  3.2±1.3 | NR  NR | 6.3±0.4  8.1±0.3 | 6.0±1.5  6.8±1.4 | 2.0±0.4  2.3±0.5 | 1.7±0.4  2.1±0.6 | 82.9%  35.3% | A1C <6.5% without anti-diabetes medications. |
| **Summary** | **SG-TB**  **SG** | 9.95  4.01  p<0.05 | 9.4±3.4  7.0±2.5  p<0.05 | 8.5±1.7  6.6±1.3 p<0.05 | 3.1±1.2  4.0±1.4p<0.05 | 80.9%  11.8%  p<0.05 | 5.5±1.1  5.4±1.2  p=0.33 | 5.7±1.2  5.5±1.2  p=0.05 | **Conclusion:** Preoperative analyses revealed that patients undergoing SGTB presented with a more advanced diabetic status, characterized by significantly higher fasting plasma glucose, HbA1c, longer diabetes duration, and greater insulin use compared to the SG group. Postoperatively, no significant difference was observed in fasting glucose levels between the two procedures, while HbA1c remained slightly lower in the SG group—a finding that may reflect the persistent influence of baseline disparities rather than a true therapeutic advantage. These results underscore the importance of accounting for preoperative metabolic severity when interpreting surgical outcomes. | | | |
| **SG-TB vs. RYGB** | | |  |  |  |  |  |  |  |  |  |  |
| Wael ^[31]^ | SASI  RYGB | NR  NR | NR  NR | NR  NR | NR  NR | NR  NR | NR  NR | NR  NR | NR  NR | NR  NR | 88.9%  87.5% | FPG<100 mg/dL, A1C <6% without anti-diabetes medications. |
| Erol ^[32]^ | SASI  RYGB | 9.2±6.7  10.2±6.9 | 12.3±3.4  11.6±3.6 | 9.5±2.3  8.9±1.8 | 3.1±1.0  2.9±0.8 | NR  NR | 5.2±1.0  5.4±0.7 | 5.0±0.5  5.0±0.6 | NR  NR | NR  NR | 96.1%  95.6% | A1C <6.5% without anti-diabetes medications. |
| Ece ^[37]^ | SG-TB  RYGB | 8.2±4.4  7.8±3.6 | 10.8±1.2  10.9±1.0 | 9.0±1.2  8.9±1.4 | 5.2±1.3  4.8±1.1 | NR  NR | 5.7±0.7  5.9±0.8 | 5.4±1.1  5.5±1.0 | 3.5±1.1  3.7±1.0 | 2.2±0.9  2.1±1.2 | 77%  79.6% | FPG<100 mg/dL or A1C <6.5% without anti-diabetes medications. |
| Mahdy ^[38]^ | SASI  RYGB | NR  NR | 8±4.2  6.5±3.5 | 6.7±2.8  7.1±2.4 | NR  NR | NR  NR | 5.1±2.7  5.7±1.9 | 5.8±2.7  5.4±1.5 | NR  NR | NR  NR | 82.7%  73.7% | FPG<110 mg/dL or A1C <6.5% without anti-diabetes medications. |
| **Summary** | **SG-TB**  **RYGB** | 8.9±6.0  8.9±5.3  p=0.98 | 10.4±3.4  10.1±2.8  p=0.48 | 8.4±2.3  8.5±1.8 p=0.71 | 3.8±1.2  3.9±1.0  p=0.37 |  | 5.3±1.8  5.7±1.1  p=0.03 | 5.4±1.8  5.3±1.0  p=0.65 | **Conclusion:** Preoperative assessment demonstrated comparable baseline characteristics between SG-TB and RYGB groups across FPG, HbA1c, diabetes duration, and C-peptide levels (all p>0.05). Postoperatively, while HbA1c reduction was equivalent between procedures (p=0.65), SG-TB demonstrated superior fasting glucose control, suggesting differential effects on glucose homeostasis despite comparable overall glycemic control. | | | |

HA1C, Glycated Haemoglobin A1c; FPG, Fasting Plasma Glucose; C-pep, C-peptide; HOMA-IR, Homeostatic Model Assessment of Insulin Resistance; T2DM, type 2 diabetes mellitus; SASI, anastomosis sleeve ileal bypass; SG-TB, SG with transit bipartition, RYGB, Roux-en-Y gastric bypass; SG, sleeve gastrectomy; NR, not reported.

Supplemental Table 6. Nutritional outcomes of studies included in the meta-analysis

| Author (ref) | Study design | **Lab result**  Albumin g/L | Hypoalbuminemia | Hb g/L | Anemia | Iron  mmol/L | Calcium  mmol/L | Vit B12  pg/mL | Vit B12 Deficiency | VitD ng/mL |
| --- | --- | --- | --- | --- | --- | --- | --- | --- | --- | --- |
| **SG-TB vs. SG** | |  |  |  |  |  |  |  |  |  |
| Qin ^[30]^ | SASI  SG | 44.8±2.8  45.8±3.1 | NR  NR | 125.1±17.4  130.9±2.4 | NR  NR | **7.8±0.3**  **8.0±0.4** | **2.29±0.1**  **2.35±0.1** | NR  NR | NR  NR | NR  NR |
| Yu ^[34]^ | SASI  SG | 45.0±2.6  44.9±1.9 | NR  NR | NR  NR | NR  NR | NR  NR | **2.53±0.06**  **2.57±0.15** | 574.4±203.6  549±175.1 | NR  NR | **15.6±5.5**  **22.4±7.4** |
| Mahdy ^[39]^ | SASI  SG | **34.3±2.7**  **37±3.8** | NR  NR | NR  NR | NR  NR | NR  NR | NR  NR | **479.8±334**  **308.6±171** | NR  NR | NR  NR |
| **Summary** | Based on limited data with predominantly 12-month follow-up, comparative analysis of nutritional parameters between SG-TB and SG reveals a largely comparable safety profile regarding albumin and hemoglobin levels. However, SG may be associated with more favorable calcium and vitamin D levels postoperatively, whereas SG-TB appears to demonstrate potential advantages in maintaining serum iron and vitamin B12 levels, though the evidence remains inconsistent across studies. | | | | | | | | | |
| Wael ^[31]^ | SASI  RYGB | NR  NR | 3.0%  0% | NR  NR | 0%  5.0% | NR  NR | NR  NR | NR  NR | 3.0%  0% | NR  NR |
| Erol ^[32]^  (2 year) | SASI  RYGB | NR  NR | NR  NR | 133.9±21.2  130.4±20 | NR  NR | **8.4±2.1**  **7.1±1.9** | NR  NR | 310.4±122  305.2±119 | NR  NR | NR  NR |
| Ece ^[37]^ | SG-TB  RYGB | 44±11  43±9 | NR  NR | 135 ± 24  136 ± 21 | NR  NR | **6.9±1.7**  **4.2±1.7** | NR  NR | **386.1±39.7**  **282±75.4** | NR  NR | **30.5±9.9**  **19.4±5.6** |
| Mahdy ^[38]^ | SASI  RYGB | 33.8±7  36±13 | **NR**  **NR** | **128 ± 16.6**  **118 ± 16** | NR  NR | **12.4±6**  **7.±5.2** | NR  NR | NR  NR | NR  NR | NR  NR |
| **Summary** | In comparisons with RYGB, SG-TB shows a trend toward better preservation of iron status, vitamin B12, and vitamin D levels based on available data. While hypoalbuminemia and anemia rates were low in both groups, SG-TB demonstrated numerically lower incidence of anemia in one study. The limited reporting and short-term follow-up constrain definitive conclusions, but SG-TB appears metabolically safer than RYGB regarding micronutrient preservation. | | | | | | | | | |

Note: NR = not reported; values in bold represent statistically significant differences (p < 0.05) as reported in the original studies. Laboratory reference ranges: Albumin (35–50 g/L), Hemoglobin (Hb) (male: 130–175 g/L, female: 115–155 g/L), Iron (7–27 μmol/L), Calcium (2.1–2.6 mmol/L), Vitamin B12 (200–900 pg/mL), Vitamin D (sufficiency >20 ng/mL).

Supplemental Table 7. Summary of baseline and surgical characteristics

| Variables | Compare | Include study | MD (95%CI) | p value | I^2^ value Heterogeneity | Egger’s p value  Publication bias |
| --- | --- | --- | --- | --- | --- | --- |
| FPG (baseline) | SG-TB vs. SG | 4 (N=514) | 0.74 (-0.3-1.78) | 0.16 | 72% | 0.08 |
|  | SG-TB vs. RYGB | 3 (N=320) | 0.47 (-0.45-1.39) | 0.32 | 55.1% | 0.11 |
| HbA1C (baseline) | SG-TB vs. SG | 4 (N=514) | 0.53% (0.12-0.93%) | 0.01 | 11.8% | 0.35 |
|  | SG-TB vs. RYGB | 3 (N=320) | 0.17% (-0.29-0.63%) | 0.48 | 16.1% | 0.81 |
| C-peptide (baseline) | SG-TB vs. SG | 3 (N=453) | -0.08 (-1.52-1.37) | 0.91 | 96% | 0.62 |
| Operation time | SG-TB vs. SG | 4 (N=315) | 30.4 (8.6-52.2) | 0.006 | 96% | 0.89 |

OR, odd ratio; CR, complete remission; MD, mean difference; CI, confidence interval; A1C, Glycated Haemoglobin A1c; FPG, Fasting Plasma Glucose; C-pep, C-peptide; SG-TB, SG with transit bipartition, RYGB, Roux-en-Y gastric bypass; SG, sleeve gastrectomy

Supplemental Table 8. Characteristics of surgery

| Author | Patients n | Description | Small bowel length (cm) | BP length (cm) | Common channel length (cm) |
| --- | --- | --- | --- | --- | --- |
| Qin ^[30]^ | SASI, | BP limb: 50%–60% of the total length of the small intestine from Treitz ligament  Common channel: >350cm | 768±110.5 | 377.5±76.1 | 390.5±66.8 |
| Wael ^[31]^ | SASI | BP limb: not mentioned  Common channel: 250–300cm | NR | NR | NR |
| Sewefy ^[43]^ | SASI  SASJ | Common channel: 250cm from ileocecal junction  BP limb: 1/3 of the length from Treiz Ligament | 744±231  740±120 | NR  NR | NR  NR |
| Erol ^[32]^ | SASI, | BP limb: not mentioned  Common channel: 300cm | NR | NR | NR |
| Foschi ^[33]^ | SASI | BP limb: not mentioned  Common channel: 350cm | NR | NR | NR |
| Yu ^[34]^ | SASI | BP limb: not mentioned  Common channel: 260cm | NR | NR | NR |
| Demir ^[44]^ | SASI  SG-TB | BP limb: not mentioned  Common channel: 300cm  Gastroileostomy: 250cm to ileocecal junction  Common channel: 100cm | NR  NR | NR  NR | NR  NR |
| Hosseini ^[45]^ | SASI  SASI-Braun | BP limb: not mentioned  Common channel: 1/3 of the length from ileocecal junction  Braun: 30cm from gastroileal anastomosis on each limb | NR | NR | NR |
| Yildirak ^[35]^ | SASI | BP limb: not mentioned  Common channel: 300cm | NR | NR | NR |
| Kirkil ^[36]^ | SG-TB | Gastroileostomy: 300cm to ileocecal  Common channel: 150cm | NR | NR | NR |
| Hosseini ^[46]^ | SASI  SASJ | Common channel: 1/3 of the length from ileocecal junction  BP limb: 1/3 of the length from Treiz ligament | 744±231  744±231 | NR  247±73 | 293±62  NR |
| Ece ^[37]^ | SG-TB | Gastroileostomy: 230cm to ileocecal  Common channel: 80cm | NR | NR | NR |
| Mahdy ^[38]^ | SASI | BP limb: not mentioned  Common channel: 300cm | NR | NR | NR |
| Mahdy ^[39]^ | SASI | BP limb: >200cm  Common channel: 300cm | NR | NR | NR |
| Widjaja ^[47]^ | SG-TB  SASI-Braun | Gastroileostomy: 260cm to ileocecal  Common channel: 220cm  Common channel: 260cm  Braun: 30 cm from gastroileal anastomosis on each limb | NR | NR | NR |
| Madyan ^[40]^ | SASI | BP limb: not mentioned  Common channel: 300cm | NR | NR | NR |
| Emile ^[41]^ | SASI | BP limb: not mentioned  Common channel: 250cm | NR | NR | NR |
| Topart ^[48]^ | SG-TB | Gastroileostomy: 250cm to ileocecal  Common channel: 100cm | NR | NR | NR |
| Arslan ^[49]^ | SG-TB  SASI | Gastroileostomy: 250cm to ileocecal  Common channel: 100cm  BP limb: not mentioned  Common channel: 300cm | NR | NR | NR |
| Yormaz ^[42]^ | SG-TB | Gastroileostomy: 150cm to ileocecal  Common channel: 80cm | NR | NR | NR |

SASI, anastomosis sleeve ileal bypass; SASJ, single anastomosis sleeve jejunal bypass, SASI-B, SASI with Braun anastomosis; SG-TB, SG with transit bipartition, RYGB, Roux-en-Y gastric bypass; SG, sleeve gastrectomy; BPD-DS, Biliopancreatic Diversion-Duodenal Switch; EWL, excess weight loss; TWL, total weight loss; NR, not reported; BP, Biliopancreatic.

Supplemental Table 9. Newcastle Ottawa Quality Assessment for the Included Studies

| Author (ref) | 1-Representativeness of the Exposed Cohort? *=Truly or somewhat representative | 1-Selection of the non-exposed? *= Drawn from the same community | 1-Ascertainment of exposure? *=Secure record or structured interview | 1-Demonstration That outcome was not present at the start of the study? *= Yes | 2-Comparability based on design or analysis controlled for confounders *=Yes | 3-Assessment of outcome. *=Independent assessment or record linkage | 3-Was follow-up long enough for outcomes to occur? *=Yes | 3-Adequacy of follow-up of cohorts *=No loss of follow-up of patients (No attrition bias) | Result |
| --- | --- | --- | --- | --- | --- | --- | --- | --- | --- |
| Qin ^[30]^ | * | * | * | * | * | * | * | * | Good |
| Wael ^[31]^ | * | * | * | * |  | * | * | * | Fair |
| Erol ^[32]^ | * | * | * | * |  | * | * | * | Fair |
| Foschi ^[33]^ | * | * | * | * | * | * | * | * | Good |
| Yu ^[34]^ | * | * | * | * | * | * | * |  | Fair |
| Yildirak ^[35]^ | * | * | * | * | * | * | * | * | Good |
| Kirkil ^[36]^ | * | * | * | * | * | * | * | * | Good |
| Ece ^[37]^ | * | * | * | * | * | * | * | * | Good |
| Mahdy ^[38]^ | * | * | * | * | * | * | * | * | Good |
| Mahdy ^[39]^ | * | * | * | * | * | * | * | * | Good |
| Madyan ^[40]^ | * | * | * | * |  | * | * |  | Fair |
| Emile ^[41]^ | * | * | * | * | * | * | * | * | Good |
| Yormaz ^[42]^ | * | * | * | * |  | * | * |  | Fair |
| Sewefy ^[43]^ | * | * | * | * | * | * | * | * | Good |
| Demir ^[44]^ | * | * | * | * |  | * | * |  | Fair |
| Hosseini ^[45]^ | * | * | * | * |  | * | * |  | Fair |
| Hosseini ^[46]^ | * | * | * | * | * | * | * | * | Good |
| Widjaja ^[47]^ | * | * | * | * |  | * |  | * | Fair |
| Topart ^[48]^ | * | * | * | * | * | * | * | * | Good |
| Arslan ^[49]^ | * | * | * | * |  | * |  |  | Fair |

(1=Selection domain, 2=Comparability domain, 3=Outcome/exposure domain. Good quality: 3 or 4 stars in selection domain AND 1 star in comparability domain AND 2 or 3 stars in outcome/exposure domain; Fair quality: 2 stars in selection domain AND/OR 0 or1 stars in comparability domain AND 2 or 3 stars in outcome/exposure domain; Poor quality: 0 or 1 star in selection domain OR 0 stars in comparability domain OR 0 or 1 stars in outcome/exposure.)
